# Emergency Medical Services Clinicians’ Pediatric Destination Decision-Making: A Qualitative Study

**DOI:** 10.7759/cureus.17443

**Published:** 2021-08-25

**Authors:** Kyle A Fratta, Jennifer N Fishe, Ellen Schenk, Jennifer F Anders

**Affiliations:** 1 Emergency Medicine, University of Pittsburgh Medical Center, Harrisburg, USA; 2 Pediatric Emergency Medicine, University of Florida College of Medicine, Jacksonville, USA; 3 Epidemiology and Public Health, Johns Hopkins Bloomberg School of Public Health, Baltimore, USA; 4 Pediatric Emergency Medicine, Johns Hopkins University School of Medicine, Baltimore, USA

**Keywords:** paramedic, emergency medical technician, emergency medical service, pediatrics emergency, ems

## Abstract

Objective

This study sought to identify factors that influence emergency medical services (EMS) clinicians’ destination decision-making for pediatric patients. We also sought EMS clinicians’ opinions on potential systems improvements, such as protocol changes and the use of evidence-based transport guidelines.

Methods

Thirty-six in-depth phone interviews were conducted using a semi-structured format. We utilized a modified Grounded Theory approach to understand the complicated decision-making processes of EMS personnel. Memo writing was used throughout the data collection and analysis processes in order to identify emerging themes. The research team utilized hierarchical coding of interview transcripts to organize data into sub-categories for final analysis.

Results

EMS clinicians cited the perceived need for specialty care, the presence of a medical home, a desire for improved continuity of care, and the availability of aeromedical transport as factors that promoted transport to a pediatric specialty center. They voiced that children with emergent stabilization needs should be transported to the closest facility, however, they did not identify any specific medical conditions suitable for transport to non-specialty centers. EMS clinicians recommended improvements in pediatric-specific education, improved clarity of hospitals’ pediatric capabilities, and the creation of a pediatric-specific destination decision-making tool.

Conclusion

This study describes specific factors that influence EMS clinicians’ transport destination decision-making for pediatric patients. It also describes potential systems and educational improvements that may increase pediatric transport directly to definitive care. EMS clinicians are in support of specific designations for hospitals’ pediatric capabilities and were in favor of the creation of a formal destination decision-making tool.

## Introduction

Emergency medical services (EMS) clinicians (Basic and Advanced Emergency Medical Technicians and Paramedics) are tasked with assessing, treating, and transporting patients in the prehospital environment [1-3]. For pediatrics, the transport destination determined by the EMS clinician is a particularly important aspect of the child’s overall emergency care. If a child is transported to a hospital that is not capable of providing definitive care, they must undergo interfacility transport (IFT) to another facility (“secondary transport”) which both prolongs care and is associated with patient harms [4-9].

While direct transport protocols proliferate in adult populations such as ST-elevation myocardial infarction (STEMI), stroke, and psychiatric emergencies, similar guidelines and decision-making tools have not taken hold for pediatric patients with the exception of major trauma [7,10,11]. In the absence of established evidence-based guidelines (EBGs), EMS clinicians rely on their gestalt to determine the appropriate destination decision for pediatric patients. While some published literature exists regarding EMS pediatric transport patterns, little is known about how EMS clinicians make these decisions [12-14].

At the same time, there is increasing regionalization of pediatric-specific services away from community hospitals and to large tertiary care centers [15-17]. The regionalization of pediatric-specific resources necessitates that EMS agencies stay abreast of their local facilities' pediatric-specific capabilities. However, the confusing nature of which pediatric services are provided at which hospitals makes the destination decision difficult and introduces the possibility of under-triage (transporting children who require specialty services to a non-specialty hospital) and over-triage (transporting children to a specialty center unnecessarily) [13].

EMS clinicians have historically reported discomfort with treating pediatric patients [18-21]. Previous studies have found infrequent exposure to pediatric patients and limited pediatric-specific formal education lie at the heart of that discomfort [18-20,22,23]. Additionally, there is even less exposure to critically ill children and in performing advanced life support (ALS) skills and procedures [19].

As a foundation towards building evidence-based guidelines for the pediatric transport destination decision-making process, understanding EMS clinicians’ current decision-making process is essential. Therefore, this study was designed to examine factors related to EMS clinicians’ pediatric destination decision-making, including those that encourage transport to a pediatric specialty center as well as transport to local non-specialty facilities. We also assessed EMS clinicians’ opinions of ways to improve destination decision-making for pediatric patients.

## Materials and methods

Study design

This exploratory study utilized a modified Grounded Theory approach for data collection and analysis in order to understand the complicated decision-making processes of EMS personnel in determining transport destinations for pediatric patients [24]. We defined the research questions presented in Appendix A a priori but allowed for new themes and questions to emerge as the data collection and analysis progressed. Grounded Theory has been used previously in qualitative EMS systems research including both protocol implementation and pediatric-specific clinical research [25-27]. The Johns Hopkins Medicine Institutional Review Board (IRB) approved this study. Each informant provided oral consent to be interviewed and was audio recorded as part of the study. The research team kept the audio recordings, transcripts, and names of the informants confidential throughout the study, accessible only to the researchers.

Sampling and study data

Study participants were solicited from an online survey of EMS clinicians in Maryland conducted in June-August 2015. One survey question asked respondents whether or not they would be willing to volunteer for a follow-up phone interview in exchange for a $20 gift card. Among those who volunteered for the phone interview, participants were randomly selected by the researchers within three targeted demographic groups as self-identified on the online survey: clinician certification level, geographic location, and employment status. The clinician levels included were ALS, such as Advanced Emergency Medical Technicians (EMTs) and paramedics, and Basic Life Support (BLS), such as Basic EMTs and Emergency Medical Responders. Geographic location was defined by whether the participant identified his or her primary EMS station as being in a rural or urban area, and employment status consisted of whether the participant’s primary EMS role was on a career or volunteer basis. Personnel were interviewed within each of the demographic sub-categories until thematic saturation within each of the six groups was reached. 

Study protocol

Data were collected via a recorded telephone interview with verbal consent provided by participants. For each in-depth interview (IDI), the research team explained to the participant the study purpose, risks, and benefits. The research team created an interview guide to loosely structure the initial IDI and revised the guides as needed to best clarify questions and based on the identification of emerging themes. The guide for the IDIs provided a semi-structured format for exploring informants’ experiences with and options for transporting pediatric patients in his or her jurisdiction, thoughts on the utility of creating a medical pediatric triage tool, as well as transport decisions on four hypothetical clinical scenarios created by the research team (Appendix A). The four clinical scenarios were intended to supplement the questions on the informant’s experiences and designed to further elucidate the informant’s decision-making process with the presentation of specific clinical details that the informant may not have recalled from his or her own transport experiences. The researchers took brief hand-written notes during each interview to document the tone. Themes were recorded for the adaptation of future IDIs and the research team transcribed the audio recording of each interview verbatim for analysis.

Data analysis

The multi-staged coding process began with line-by-line coding of transcribed interviews in order to identify potential themes. The research team then used a collaborative group process of focused coding to combine and or re-arrange the original list of codes as needed. Furthermore, we used a collaborative process of axial coding to create a codebook with the following overarching axes: 1) Factors influencing transport decisions, 2) Utilization of triage tools. Based on the codebook, the research team coded each transcript using Atlas.ti version 7 (Scientific Software Development, Berlin, Germany). Lastly, the research team used hierarchical coding to organize data into sub-categories. 

## Results

This study utilized qualitative data collected between August and October 2015 from 36 in-depth phone interviews with EMS personnel in Maryland. The duration of the interviews ranged from 25 minutes to 90 minutes, with an average length of 40 minutes. Among the 36 participants, 25 were male and 11 were female, 19 were ALS and 17 were BLS clinicians, 14 worked in rural locations and 22 worked in urban locations, and 21 were career and 15 were volunteer clinicians (Table 1).

**Table 1:** Characteristics of in-depth interview participants

		Number of clinicians		Number of clinicians
Clinician Level	ALS	19	BLS	17
Geographic Location	Urban	22	Rural	14
Employment status	Career	21	Volunteer	15
Gender	Male	25	Female	11

Factors that promote transport to a pediatric specialty center

We identified five factors that promoted EMS transport to a pediatric specialty center. These factors, a summary of the overall themes, and representative quotations are presented in Table 2. Clinical factors included perceived medical need for specialty care, the presence of a medical home or previously established care at a specific facility, and the desire for improved continuity of care. Operational factors included the availability of helicopter-EMS (HEMS).

**Table 2:** Factors that promote transport to a pediatric specialty center EMS: Emergency medical services; HEMS: Helicopter-EMS

Factor	General Theme	Specific Quotes
Medical Home	If a child has a pre-existing medical condition, technology dependence, or established specialty care at a specific hospital then EMS clinicians would prefer to transport the child to that hospital	“I would aim for where this child is being followed. Just because of the prior relationship with the hospital I think it would make everybody happier. The following physician, the family I think they would prefer me as a paramedic to transport to the facility where the child has a history.”
		“If they are being seen at [a pediatric specialty center] for some specific reason we try to make every accommodation to take them to [their home center] so they can be seen at that level of care.”
Perceived Need for Specialty Care	If a child has a significant injury or illness that the EMS clinician believes will require specialty care they would prefer to transport the child to a pediatric specialty center	“Based on my experience…he's probably going to need surgery so he's not going to any of the local facilities. He's going to go to the specialty center.”
		“With this patient still being conscious I’d be looking to try to get him as quick as I could to the specialty pediatric center.”
Continuity of Care	EMS clinicians want to minimize delays in patient care and logistical delays/inconveniences to family members/caregivers	“If we take our patient to a facility where they are going to have to be transported out of, then it’s just delaying the definitive care for that patient. It is not in our or the patient’s best interest. Because in the long term we just want to get peds to more capable definitive care facility where they will get taken care of.”
		“It would be nice for him to go to the ultimate destination where he has continuance of care.”
Availability of HEMS	EMS clinicians who practice in rural areas are more likely to transport to a pediatric specialty center if HEMS is available to expedite the transport time	“He would be going to the [specialty center], more than likely due to the transport time I would like to utilize aviation.”
		“If I thought I can maintain his airway I absolutely would try to get him to the place the specialty center and that would probably be an aviation call.”

Factors that promote transport to local non-specialty facilities

We identified four factors that promoted EMS transport to local non-specialty facilities. These factors, a summary of the overall theme, and representative quotations are presented in Table 3. Clinical factors included critically ill patients and EMS discomfort in treating pediatric patients. Operational factors included long transport times to specialty centers.

**Table 3:** Factors that promote transport to a local non-specialty center EMS: Emergency medical services; BLS: Basic life support

Factor	General Theme	Specific Quotes
Critically Ill Children	EMS clinicians are able to identify critically ill children and understand that these patients need emergent stabilization at the nearest emergency department before interfacility transport for definitive care	“Overall, if the child is sick, and might be getting worse, I would feel more comfortable transporting this child to a local facility where they can stabilize this patient and then transfer to a specialty center.”
		“The closest hospital isn’t likely to have the resources the patient needs [for definitive care]. Based on his [clinical condition] I don’t want to take an extra 15 minutes to go to the specialty center. They can stabilize him if necessary and transfer when appropriate.”
EMS Discomfort with Pediatric Patients	EMS clinicians, particularly of BLS certification, have a general discomfort in caring for children and are more likely to consult, call for a higher level of care, or transport to a nearby non-specialty facility especially with children with serious and/or preexisting conditions	“It would be nice for him to go to [specialty center]. But is he stable enough to make that run in an ambulance? …I think I don't want to be in the ambulance for 45 minutes given the set of vitals [in this scenario]. I’d be calling for help.”
		“Pediatric patients always make me consult more frequently, I'm always a little more nervous about pediatrics than adults.”
Long transport times to a pediatric specialty center	EMS clinicians factor total transport time into their destination decision making both from a clinical and operational standpoint	“I know it seems like it’s a very serious situation. He needs to be seen sooner than you can get to the specialty center. I think 30 minutes is too long.”
		“Anything over 30 minutes. That would definitely factor for this patient. I don’t want him in the back of my ambulance for more than a half hour.”

Other factors that weigh into destination decision-making

We identified three other factors that EMS clinicians cited as being important in their destination decision making, but did not specifically align with transport to either a pediatric specialty center or a local non-specialty center. These factors, a summary of the overall theme, and representative quotations are presented in Table 4. These included family/caregiver destination preference, online medical direction, offline EMS clinician protocols, and confusion over specific hospitals’ pediatric capabilities.

**Table 4:** Other factors that weigh in EMS destination decision making

Factor	General Theme	Specific Quotes
Family/caregiver destination preference	Emergency medical services (EMS) clinicians take the family/caregiver’s preferences into consideration when determining a transport destination regardless of the specific facilities’ pediatric capabilities	“Based on the medical issue and the severity, if the patient’s family asks to go to a specific hospital, if its within reason, we will try and accommodate.”
		“We have some parents who insist on going to other hospitals because they are more comfortable with the hospital.”
Online medical direction	EMS clinicians regularly utilize online medical consultation with both local non-specialty facilities and pediatric specialty centers to allow for improved destination decision-making	“Do you want me to bring the patient to the local hospital to stabilize before they get transported on? And then I let the hospital tell me yes or no. But at least I would ask that question.”
		“I would definitely be on the radio with that hospital.”
Offline EMS clinician protocols	EMS clinicians utilize standing off-line medical direction in determining an appropriate destination decision	“Medical cases have a different protocol. We have to take them to the closest emergency department.”
		“…but unfortunately, protocol states we have to take them to the closest facility.”
Confusion over specific hospitals’ pediatric capabilities	EMS clinicians expressed some confusion over which hospital offered pediatric services and over which specific services are available at any given pediatric center	“[The general public] does not know that you don’t have a pediatric facility on site. If their kid is sick, they are going to bring that kid to that hospital. But it’s also EMS trying to find out what hospitals are capable of doing what.”
		“I hate to tell you this, but I didn't get a memo. I was not made aware that they were taking pediatrics.”

Suggestions for improved pediatric destination decision-making

EMS clinicians offered several suggestions in order to better facilitate appropriate destination decision making for pediatric patients. These suggestions are outlined in Table 5. They included clinical improvements in pediatric-specific education as well as the creation of a pediatric-specific triage tool. Systems improvement suggestions included revision of offline EMS clinician protocols, improved clarity of hospital’s pediatric capabilities, and guidance for medically complex pediatric patients or those with medical homes. 

**Table 5:** Suggestions for improved EMS clinician pediatric destination decision-making

Suggestion	General Theme	Specific Quotes
Improvements in pediatric specific education	Emergency medical services (EMS) clinicians would like more pediatric specific clinical and transport-need education both during initial certification and during continuing medical education classes	“Education is extremely warranted for pediatrics. [EMS clinicians] need to be reeducated and reeducated and reeducated on how to deal with pediatrics - trauma and medical.”
		“I think it’s just education that would be helpful for the EMS providers. I mean we treat adults and the elderly all the time and I don’t feel like we get enough pediatric education to know to be prepared to take care of them.”
Creation of a pediatric specific triage tool	EMS clinicians would like formal guidance in destination decision making for both medical and trauma pediatric patients	“Well I think we really need to have [a tool], it would be nice to see a pediatric decision tree for medical as well as trauma. Just for peds.”
		“It would be good to have a tool and you can follow to make sure you are getting the patient to the right place.”
Revision of offline EMS clinician protocols	EMS clinicians would like offline medical protocols to include specifics of facilities’ pediatric capabilities, specifications for on-line medical direction, and for protocol to allow for bypassing closer non-specialty facilities when medically appropriate	“Unfortunately, protocol States we have to take them to the closest facility. If they would alter the pediatric protocol to say for a child which is stable enough to transport them to the closest pediatric hospital emergency room it would be no problem”
Improved clarity of hospital’s pediatric capabilities	EMS clinicians would like explicit information on the pediatric capabilities of specific hospitals	“I think making sure that the current capabilities of any facility is outlined more explicitly in the state EMS protocols. So that [EMS Clinicians] can be more comfortable with transport decisions.”
		“Training classes and recertification classes about what facilities we have available to us that have pediatric capabilities… I think that’s very important.”
Guidance for medically complex pediatric patients or those with a medical home	EMS clinicians would like more formal guidance on where to transport patients with complex medical histories, technology dependence, and/or medical homes	“I tend to like if it’s a specialty patient, I’d rather take them to the place where they had it done, the place where they know the patient. I’d just call the center and be like hey we’ve got this patient here and this is what’s going on. What do you want us to do?”
		“Special needs populations with children, where they have past medical history…Maybe they’re potentially stable now, but do they fit in any category that should go to the specialty center right away, rather than getting evaluated locally?”

## Discussion

This study aimed to elucidate key factors related to EMS clinicians’ pediatric destination decision-making. A secondary aim was to highlight potential systems and educational changes that would improve pediatric destination decision making. The results outline several factors that prompt EMS clinicians to transport children to pediatric specialty facilities, however, we discovered a dearth of specific factors that prompted the transport of children to local non-specialty facilities. The EMS clinicians interviewed also provided several tangible recommendations to improve pediatric destination decision-making.

The EMS clinicians interviewed in this study repeatedly communicated their preference to transport children directly to an appropriate center whenever feasible. They cited the perceived need for specialty care as a motivating factor to choose direct transport to a pediatric specialty center. The respondents also cited a desire for continuity of care among pediatric patients. Several specifically mentioned a desire to transport a child to their medical home. This is particularly important as children with special healthcare needs (CSHCN) have a disproportionately high rate of EMS usage and more frequently require IFT [28,29]. Despite a general discomfort in providing care to pediatric patients, EMS clinicians understand the needs of both general pediatrics and CSHCN. That clinical intuition can be refined in formal education and incorporated in future EBGs. As a part of this education, EMS clinicians should be introduced to the Emergency Information Form (EIF), a simple and easy to utilize document for CSHCN endorsed by the American Academy of Pediatrics and the American College of Emergency Physicians [3,30]. If the population of children who need a pediatric capable destination is successfully identifiable by EMS clinicians and direct transport is possible, this could relieve a significant burden on local non-specialty facilities as well as patients and their families.

EMS clinicians identified only one factor that specifically promoted transport to local non-specialty facilities - children that were critically ill and required emergent stabilization. Other factors listed as promoting transport to local non-specialty facilities included EMS discomfort in caring for children and confusion over receiving hospitals’ pediatric capabilities. Both of those items represent educational and operational shortcomings more so than physiologic/clinical indication for transport. Few clinicians interviewed were able to identify specific conditions that local non-specialty facilities can adequately address. While it is important in developing EBGs and transport protocols to consider acuity and complex conditions, equal emphasis should be placed on triaging children to local non-specialty or modestly capable pediatric centers when appropriate. Thus, that area represents a concrete area for improved EMS research and education.

Several operational considerations also influenced EMS transport decision-making. Predictably, those in rural settings more often cited transport time as contributing factor when transporting children to local non-specialty facilities or for utilizing HEMS for transport to a pediatric specialty center. While this input from our respondents was not unexpected, it underscores that future decision-making tools and transport protocols should take these operational issues into consideration, understanding that no EMS system is identical in either geography or resources. While clinical components and hospital capabilities may be standardizable, future decision-making tools should allow for individual EMS systems to input resource utilization specifics. 

A general discomfort in treating children, especially critically-ill children, was a recurring theme in the IDIs. That discomfort and the need for pediatric-specific EMS education have been reported over the past thirty years [18-22]. The IDIs revealed that despite those repeated calls for prehospital pediatric education, current educational practices are still not alleviating EMS clinicians’ widespread discomfort in caring for children. That issue will likely persist and potentially become more severe as pediatric hospital resources continue to become more centralized necessitating longer transport time to specialty care.

EMS clinicians also expressed variable levels of knowledge over hospitals’ pediatric-specific capabilities. That knowledge varied from not knowing that a given hospital even accepted children to knowing specific hours when a pediatrician was on staff. Without any widely operative and standardizable way of classifying hospitals’ pediatric-specific capabilities, the onus falls to individual EMS agencies and clinicians and requires significant experience in a geographic area of practice. During the IDIs, respondents requested specific information about their local hospitals’ pediatric capabilities. This would allow for transparency of pediatric-specific capabilities and would also allow for easier destination decision-making in the prehospital arena. Respondents also requested explicit guidance on what conditions to transport to pediatric specialty centers versus local non-specialty hospitals.

The EMS clinicians included in this study were overwhelmingly in favor of the creation of a destination decision-making tool. The minority of study participants who believed that a destination decision tool was unnecessary stated that in their local practice all pediatric medical patients are transported to the closest ED. While such practices may be appropriate in more austere geographic areas, pediatric patients should be assessed and transport destination determined on a case-by-case basis by EMS clinicians and the transport destination should be tailored to best serve the patient and their family/caregivers. The clinicians in favor of a destination decision-making tool requested that such a tool include both medical and traumatic conditions, that hospitals be differentiated by their pediatric-specific resources, and that EMS clinicians are educated as to which specific patient conditions require higher levels of pediatric specialty care. Such a decision-making tool would ideally maximize the number of patients transported to a facility capable of providing those children definitive care while optimizing scarce EMS system and hospital resources.

There are limitations inherent to interview-based qualitative research including this study. First, less than 20% of those who completed the initial online survey volunteered for the phone interview and were able to be contacted to conduct an interview. Those who volunteered for the phone interview may be particularly passionate about pediatric issues and may not represent the entire population of EMS personnel in Maryland. Second, there is the possibility of a Hawthorne effect during the interviews, particularly with regards to giving answers to the clinical scenarios. To mitigate the impact of that bias, the researchers used the scenarios to glean further details of EMS personnel’s decision-making processes, rather than the absolute number or percentage of informants who chose one destination or trauma category over another.

## Conclusions

This study describes specific factors that influence EMS clinicians’ transport destination decision-making for pediatric patients, as well as potential systems and educational improvements targeted at increasing pediatric transport to definitive care. The pediatric prehospital destination decision-making process is extremely complex and is coupled with widespread discomfort among EMS clinicians in caring for and transporting children. Improved strategies are needed for both pediatric EMS education and for the EMS destination decision-making process in order to provide quality prehospital pediatric care in an era of increasing pediatric hospital resource regionalization. EMS clinicians are in support of the creation of a formal pediatric destination decision-making tool.

## Appendices

APPENDIX A: In-Depth Interview Guide

Interview Questions: Hello. My name is XX and I am calling from Johns Hopkins I am trying to reach (name of scheduled participant). Am I speaking with the right person?  

I am calling in regards to the phone interview that I emailed you about recently, for which you volunteered via the corresponding online survey a few weeks ago. Is this still a good time for you to participate in the phone interview?

Thank you for your willingness to participate in this interview.

I need to let you know I am beginning to record this interview from this point forward. Before we get started, I would like to remind you that your completion of this telephone interview is voluntary. Your name will not be connected to any of the answers you give today. The recording and transcript of the interview will be stored in a secured location, accessible only to the members of the research team. If you wish to withdraw your consent and have your answers erased, you can tell me that at any point in the interview today. You can also skip any questions you prefer not to answer. Do you consent to continue with the interview and to be recorded?

EXPERIENCES TRIAGING PEDIATRIC PATIENTS

First, I’d like to ask about your experiences triaging pediatric patients… this information will be used to improve existing triage tools as well as to create new tools as necessary.

1. How many pediatric patients do you transport on average in a month? And, how many would you estimate are trauma versus medical cases?

2. Within the last month, what proportion of pediatric patients would you estimate that you transported to a pediatric trauma center? To a pediatric specialty center?

3. What are some of the factors that go into your decision-making process of whether to transport a pediatric patient to a pediatric facility, based on some examples of cases you have transported?

ATTITUDES TOWARD EXISTING TRIAGE TOOLS

1. How useful do you think the trauma triage tree that is included in Maryland protocols is for making trauma patient destination decisions? Tell me more about why you think it is useful/not useful.

2. How useful is the trauma triage tree is for making PEDIATRIC trauma patient destination decisions? Tell me more about why you think it is useful/not useful.

3. What are some examples of clinical situations for which you think the TTT is helpful for pediatrics?

4. Can you tell me about a clinical experience with a pediatric trauma patient where you were not sure about the best destination choice? How useful was the TTT in that instance? Why was it useful/not useful?

5. Based on your experience, what do you think should be changed about the TTT to make it more useful?

6. How helpful do you think it would be to have a pediatric triage tool for medical patients, similar to the trauma triage tree, to guide destination choices specifically for pediatric medical patients? Tell me more about why you think it would be helpful/not helpful. If helpful, follow up: For which pediatric clinical scenarios would it be helpful to have such guidance?  Can you tell me about a time that you had to make a difficult decision about a medical pediatric patient where you were not sure about the best destination choice?

SCENARIO DECISION-MAKING QUESTIONS For the next few questions, we will use the scenarios provided in the email you received.

Scenario one: •    3-year-old fell while running. Has obviously deformed right femur, pulse, and sensation intact •    Crying, complains of pain in leg •    No other injuries •    VS: HR 138, RR 28, BP 108/78, Pulse Ox 99% •    PMH, Allergies: none •    Prehospital care includes: IV start, morphine 2 mg IV

1. What trauma category would you assign this patient? (expected answers: N/A (not a trauma patient), Alpha, Bravo, Charlie, Delta, Not sure)

2. How did you make that category choice? If the participant used the trauma triage tree (TTT) to make the choice, follow up …What indicators on the TTT led you to make the choice of Category (insert response)?

3. Which destination would you choose for this patient? (options: Closest hospital, regional hospital that has a pediatrician, adult trauma center, pediatric trauma center)

4. How did you choose (insert answer 3 here) as the destination?

5. If the respondent states they used the TTT to determine destination, ask follow up; Do you agree with the trauma triage tree recommendation for destination?  And, why or why not?

6. How far away by ground ambulance would the pediatric trauma center need to be for you to feel it would be necessary to request aviation for this patient?

Follow up: Other than the travel time, are there any other factors that would have influenced your decision to request aviation for this patient?

7. How much farther away would the pediatric trauma center need to be relative to the adult trauma center for you to decide to take the patient to the adult trauma center?  What if any other factors would influence your decision?

Ok, I will be asking similar questions for the other scenarios… Scenario two: •    13-year-old boy with rolled ATV; found unconscious by family but responsive on EMS arrival. •    Bleeding from forehead laceration, bleeding from mouth, broken teeth. •    Pain in right arm/elbow. No obvious deformity. Large abrasion over forearm. •    Pain in abdomen. •    Awake but dazed, replies to questions but slow to answer. (GCS 15) •    VS: HR 118, RR 20, BP 138/78, Pulse Ox 99% •    PMH, Allergies: none •    Prehospital care includes: IV start, IV morphine dose of 4 mg

1. What trauma category would you assign this patient? (expected answers: N/A (not a trauma patient), Alpha, Bravo, Charlie, Delta, Not sure)

2. How did you make that category choice? If the participant used the trauma triage tree (TTT) to make the choice, follow up …What indicators on the TTT led you to make the choice of Category (insert response)?

3. Which destination would you choose for this patient? (options: Closest hospital, regional hospital that has a pediatrician, adult trauma center, pediatric trauma center)

4. How did you choose (insert answer 3 here) as the destination?

5. If the respondent states they used the TTT to determine destination, ask follow up; Do you agree with the trauma triage tree recommendation for destination?  And, why or why not?

6. How far away by ground ambulance would the pediatric trauma center need to be for you to feel it would be necessary to request aviation for this patient?

Follow up: What if any other factors would have influenced your decision to request aviation?

7. How much farther away would the pediatric trauma center need to be relative to the adult trauma center for you to decide to take this patient to the adult trauma center?  What if any other factors would influence your decision?

Scenario three: •    6-year-old found hanging from rope on swing set, unconscious and blue •    Now responsive with GCS 14 •    Patient has labored and difficult breathing •    Bruising to neck •    VS: HR 100, RR 18, BP 95/63, Pulse Ox 100% •    Prehospital Care: Placed on NRB with 10L oxygen

1. What trauma category would you assign this patient? (expected answers: N/A (not a trauma patient), Alpha, Bravo, Charlie, Delta, Not sure)

2. How did you make that category choice? If the participant used the trauma triage tree (TTT) to make the choice, follow up …What indicators on the TTT led you to make the choice of Category (insert response)?

3. Which destination would you choose for this patient? (options: Closest hospital, regional hospital that has a pediatrician, adult trauma center, pediatric trauma center)

4. How did you choose (insert answer 3 here) as the destination?

5. If the respondent states they used the TTT to determine destination, ask follow up: Do you agree with the trauma triage tree recommendation for destination?  And, why or why not?

6. How far away by ground ambulance would the pediatric trauma center need to be for you to feel it would be necessary to request aviation for this patient?

Follow up: What if any other factors would have influenced your decision to request aviation?

7. How much farther away would the pediatric trauma center need to be relative to the adult trauma center for you to decide to take the patient to the adult trauma center?  What if any other factors would influence your decision?

I have one more scenario; this is a medical patient, not a trauma patient. Scenario four: •    12-year-old with hydrocephalus for which he has had many surgeries and has a ventriculoperitoneal shunt. •    The shunt was last operated on one month ago. •    The child has been sleepy and vomiting earlier today. •    His mother was not able to arouse him at the end of a nap. •    VS: HR 54, RR 26, BP 138/90, Sats 100% on RA •    Glasgow coma scale score of 12: lethargic and aroused by painful stimulation only. •    There is a healing wound on his right scalp from recent shunt surgery, and the shunt bulb and tubing are palpable under the wound. The wound does not look infected. •    Physical exam is otherwise unremarkable. •    Prehospital care: IV start. IV fluids. 1. If the closest hospital is 10 minutes away, a regional pediatric center is 30 minutes away, and the pediatric specialty center where the child’s neurosurgeon is located is 45 minutes away, which destination would you choose for your transport? How did you choose (insert answer 3 here) as the destination?

2. What factors would make you more likely to pass the closer facilities and go to the specialty center?

3. What factors would make you more likely to transport to a local/closer facility?

4. How would aviation factor into your transport decisions for this patient?  How far away would the pediatric specialty center need to be to decide to request aviation for this patient?  What other factors would influence that decision?

WRAP-UP

1. This brings us to the end of the interview. After thinking through the scenarios and your professional experiences, do you have any final thoughts on adapting the existing trauma triage tree for pediatric patients or creating a similar triage tool for pediatric medical patients?  Is there anything I didn’t ask about that you think is important for us to know about destination decision-making for pediatric patients?

2. As we analyze the results of the interviews, other important questions may come up. Would it be alright for us to contact you at a later point if we have follow-up questions?
